# Transition-related outcomes among a cohort of patients with juvenile idiopathic arthritis

**DOI:** 10.1007/s10067-025-07317-y

**Published:** 2025-01-16

**Authors:** Laura De Nardi, Serena Pastore, Hajar Benaly, Francesco Rispoli, Ivan Giovannini, Luca Quartuccio, Salvatore De Vita, Alen Zabotti, Alberto Tommasini, Andrea Taddio

**Affiliations:** 1 University of Trieste, Trieste, Italy; 2 Institute for Maternal and Child Health IRCCS “Burlo Garofolo,” Via dell’Istria 65/1 – 34137, Trieste, Italy; 3 Rheumatology Department, “Santa Maria della Misericordia” Hospital, Udine, Italy

**Keywords:** Chronic disease, Health-related quality of life, Health transition, Juvenile arthritis

## Abstract

A major goal in juvenile idiopathic arthritis (JIA) long-term management is to ensure a successful transition to adult age. This study aims to assess transition outcomes in a group of JIA patients during their passage from pediatric to adult healthcare assistance at a single center. This is a cross-sectional study. All patients with JIA undergoing a transition from the Pediatric Rheumatology Service of the IRCCS “Burlo Garofolo” Hospital, Trieste, to the adult Rheumatology Service of “Santa Maria della Misericordia” Hospital, Udine, between 2017 and 2022, were enrolled. Clinical and laboratory data were collected. A semi-structured survey exploring patients’ satisfaction was distributed through email. Numerical variables were compared using Student’s *t*-test or Mann–Whitney test. Categorical variables were compared with Fisher’s exact test. We recruited 36 patients (26 female, 72.2%): 9 with polyarticular course JIA, 13 oligoarticular, 8 psoriatic arthritis, 3 systemic JIA, and 3 enthesitis-related arthritis. The mean age at transition was 18.6 (*Q*1–*Q*3, 18.3–19.1). JADAS-27 score significantly decreased after the transition, with a mean difference of 2.6 (*p* = 0.014). No patients were lost to follow-up, and in 8 out of 36 (22.2%), a step-up therapy was needed within the first 12 months. Among these, no correlation was found with the JIA subtype, age at onset, type of involved joints, and other variables explored. Finally, the 15 patients who answered the survey (response rate 50%) were satisfied about the transition process. This study described a real-life transition experience from pediatric to adult rheumatology care, showing good transition outcome measures, with no patients lost to follow-up and a reduction of JADAS-27 score after completing the process.

**Supplementary information:**

The online version contains supplementary material available at 10.1007/s10067-025-07317-y.

## Introduction

Transition from pediatric to adult health care is a critical time for adolescents with chronic diseases, regardless of the disease itself [1]. Adolescence is a vulnerable age, hallmarked by increased self-identity, somatic growth, and development of psychological and behavioral independence. Chronic conditions can affect physical, cognitive, social, and emotional status, with repercussions on siblings and parents as well [2]. The recurrence of disease flare-ups and the need for medications with their possible associated adverse effects are just some of the reasons why adolescents with chronic disease need mental coaching and should be carefully accompanied by their physicians through the transition to the adult age. They experience anxiety for the future and feeling different from peers can lead them to depression and feeling alone [2].

Juvenile idiopathic arthritis (JIA) is the most common childhood chronic rheumatic disease, encompassing all forms of arthritis that persist for more than 6 weeks, with onset before the age of 16, after the exclusion of other causes of arthritis [3]. JIA is an inflammatory disease affecting the joints and the skeleton in the maturation phase, which may lead to long-term joint damage and functional disability. Although JIA addresses only 0.1–0.4% of the pediatric population, nearly half of such patients will continue to have an active disease in adulthood [4].

Following transfer to adult health care, patients are well reported to worsen health-related outcomes, by increasing hospitalizations, poorly adhering to therapies, being lost at follow-up, scarcely controlling their disease, or experiencing health-related life difficulties and failing at school or work [5–7]. The transition of patients with JIA is further hampered by the absence of specific criteria for the assessment of disease activity, the lack of specific treatment recommendations for JIA adult patients, the poor adolescent-specific training by adulthood rheumatologists, and the lack of communication between pediatric and adulthood centers [8]. Therefore, operating a successful transition process is a major goal in JIA long-term management [8].

Core elements agreed upon for a successful transition process include the discussion of issues related to the transition process in advance, with periodic assessment of the transition readiness, encouraging self-management skills and self-awareness of the disease. Sharing medical information with the physicians that will care for the patients in adulthood can also help [9–14]. However, these recommendations are largely derived from expert consensus opinion, and specific outcome measures to define the success or the failure of transition interventions are so far missing. The satisfaction of the patient and the family, the perceived quality of care, self-reported measures of disease activity, and adherence to medication and appointments are the most used outcome indicators in transition studies [6]. A major gap on this matter is the difficulty to identify well in advance those adolescents at greater risk of poor outcomes, in order to catch them and to prevent long-term functional disability [6].

This study aims to assess transition-related outcomes in a group of JIA patients during their passage from pediatric to adult healthcare assistance at a single center. Potential correlations between disease relapses after transition and disease features in pediatric age have also been evaluated.

## Materials and methods

This is a monocentric observational cross-sectional study performed between January 2017 and December 2022. Eligible patients were aged between 14 and 20 years, with pediatric JIA onset initially followed at the Pediatric Rheumatology Service of the Institute for Maternal and Child Health IRCCS “Burlo Garofolo” of Trieste. Patients have been then transitioned to the Rheumatology Service at “Santa Maria della Misericordia” University Hospital, Udine, which is a town in the same geographical area, just 70 km apart from Trieste.

### Description of the transition policy at our center

**Fig. 1:**
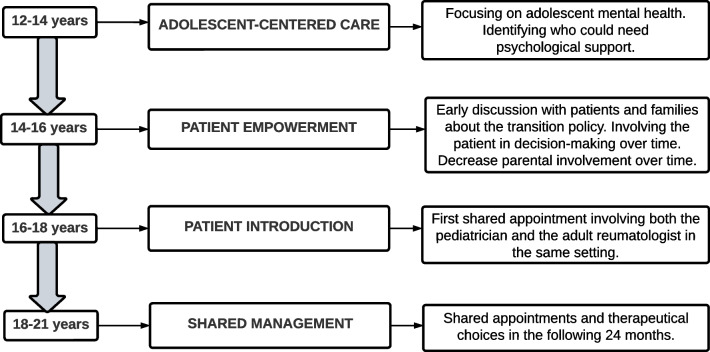
The transition model

The transition model is shown in Fig. 1. The process begins between 12 and 14 years, and it is completed approximately at the end of secondary school (usually at 18 years of age). In the beginning, the *focus* is *on specific competences of adolescent age* rather than on the future transition to adult health care. In our experience, the administration of effective medical treatment and the attention to healthy adolescent development are both associated with the success of transition. Thus, the patients are firstly asked about *the way they perceive their illness* and their treatment plans, but also their *relationships with peers* as well as their hobbies and *life expectations*. They are directly addressed by the physician, in order to explore how much JIA and the relative treatments impact their adolescence and *identify early who could benefit from a psychological support*. After the age of 14 years, the *patients and their parents are* usually *informed about the transitional policy*. As already mentioned, indeed, early discussion helps to better afford the transfer [14]. Then, the *parental involvement* is *progressively reduced* and the patient, rather than the parents, is directly addressed. In this initial phase, the patient starts to learn self-management skills about the disease and therapies, but the parents still join the visit. Later, the *parents may be asked to wait outside* and to *join the visit at the end*. This can help involve the patient in the decision-making process and prepare them for a future doctor-patient relationship. The first meeting with an adult rheumatologist usually takes place between 16 and 18 years of age. The adolescent is carefully guided and eventually transferred from the pediatric to the adult health care system (at 18–21 years of age). In this final phase, *structured appointments involve both the pediatric and adulthood physicians*, thus ensuring a proper share of medical information between clinicians and letting the patient perceive care continuity. This is an essential moment also to discuss the clinical evolution of previously transitioned patients, to consider possible problems related to the transition process and to *share therapeutical choices* that will be agreed upon *in the following 24 months*. All of this is in accordance with the clinical report from the American Academy of Pediatrics (AAP), the American College of Physicians (ACP), and the American Academy of Family Physicians (AAFP) which includes guidance for healthcare professionals regarding young patients with special health care needs [15].

### Inclusion/exclusion criteria and data collection

We enrolled all consecutive patients with an established diagnosis of JIA according to the ILAR criteria [16]. Patients with other rheumatologic conditions transitioned to the same adult care center and patients who transitioned to a different adult rheumatology center were excluded. For each patient, demographic and clinical data were collected (including age at transition, sex, age at onset, family history, number and type of involved joints, JIA category according to 2001 ILAR classification criteria [17], enthesitis, tenosynovitis or uveitis involvement). We provided all patients with a follow-up period of at least 12 months after the first visit to the adulthood rheumatologic clinic. For all patients, we recorded data about disease activity and ongoing medications, both at the time of the last pediatric visit and at the end of the follow-up period. In addition, information about the previously taken medications was gathered. We used the JADAS-27 score to evaluate disease activity on the day of examination at the pediatric center and at least after 6 months from transition; the score is produced by summing the physician’s global assessment of disease activity, the parent/patient global assessment of well-being, the assessment of active arthritis in 27 joints, and the normalized erythrocyte sedimentation rate (ESR) [18]. We considered the following outcome measures in this study, according to literature data [6]: number of patients lost during the follow-up, main increase in JADAS-27 score after transition, changes in therapies after transition, in particular, the need for scale-up therapy within the following 12 months. “Scale-up” was defined as the need to recur to an add-on or to a different therapy because the ongoing treatment did not prove to be fully effective.

Finally, just for knowledge purposes, the patients’ satisfaction rate about the transition process was evaluated by administering a Google Form with an anonymous semi-structured self-made survey sent via email, composed of a 5-point Likert scale [19] and two open questions (see Supplementary file 1). We categorized recurrent open answers and compared the results of this descriptive analysis with current literature findings. All data were collected from patient’s clinical records and stored in an anonymized Microsoft Office Excel file. Besides evaluating all the aforementioned variables, we analyzed the presence of possible associations between disease relapses after transition, with the need for a scale-up therapy, and disease features in pediatric age. The study was conducted according to the guidelines of the Declaration of Helsinki, and approved by the Institutional Review Board of IRCCS “Burlo Garofolo”—Trieste (IRB, RC 23/22, ruling no. 12, date of approval 30/12/2021).

### Statistical analysis

Continuous data were presented as means (SDs) or medians (*Q*1–*Q*3). Numerical variables were compared using Student’s *t*-test or Mann–Whitney test, after checking for normality of distribution using the Shapiro–Wilk test. Categorical variables were expressed as numbers (%) and compared with Fisher’s exact test. A *p*-value < 0.05 was considered statistically significant. Data analysis was performed using SPSS software version 26.0 (IBM).

## Results

### Patients’ recruitment and characteristics

**Fig. 2:**
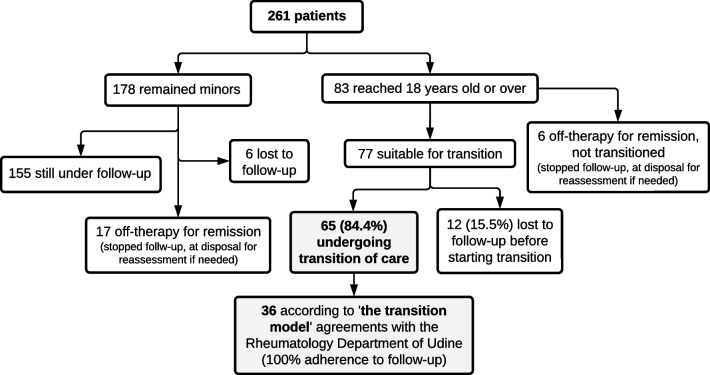
Patients with JIA cared for between 2017 and 2022

A total number of 261 individuals with JIA have been cared for at our Institute between January 2017 and December 2022 (Fig. 2). At the time of the investigation, 83 individuals came of age so they were suitable for a transition program. Six patients, who were in stable remission without treatment upon reaching 18 years of age, were not transferred but maintained contact with the center for potential needs. Sixty-five out of 83 were referred to adult rheumatology centers for transition of care. Twelve individuals (15.5%) were lost to follow-up upon reaching 18 years old and hence were not transitioned. In summary, the transition procedure was effective for 84.4% (65/77) of the individuals who could benefit from it. This study included the 36 patients (26 female, 72.2%) referred to the “Santa Maria della Misericordia” rheumatology department (Udine), with whom a more structured transition agreement exists. The transition was completed for this whole cohort according to the transition model discussed above. The main characteristics of the patients (JIA types distribution, median age at the transition, clinical features) are resumed in Table 1. On average, we performed the first transition visit in the adulthood health care setting after 114 days from the last pediatric visit (SD 132).

**Table 1:** Main characteristics of the patients

Total no. of patients	36 (100%)
Female sex, *n* (%)	26 (72.2%)
Age at onset, average (SD)	10.3 (4.19)
Age at transition, median (*Q*1–*Q*3)	18.6 (18.3–19.1)
Family history of autoimmunity, *n* (%)	6/36 (16.7%)
JIA categories^1^, *n* (%)	
Polyarticular	9/36 (25%)
Oligoarticular	13/36 (36.1%)
Extended oligoarticular subtype	7/13 (19.4%)
Psoriatic arthritis	8/36 (22.2%)
ERA	3/36 (8.3%)
Systemic	3/36 (8.3%)
ANA positivity, *n* (%)	25/36 (69.4%)
Cervical spine involvement, *n* (%)	6/36 (16.7%)
Enthesitis, *n* (%)	6/36 (16.7%)
Tenosynovitis, *n* (%)	6/36 (16.7%)
Uveitis, *n* (%)	5/36 (13.9%)

^1^JIA categories according to 2001 ILAR classification criteria

### Transition-related outcomes

Overall, JADAS-27 score values significantly decreased after the completion of the transition process, with a mean difference of 2.6 (*p* = 0.014); subgroup analysis revealed no correlation between the change in JADAS-27 score and the other variables considered. None of our 36 patients was lost during the follow-up, and such data are confirmed also in the 22/36 patients for which a longer follow-up period was available: 2 years for 12 patients, 3 years for 8 patients, 4 and 5 years, respectively, for the other 2 patients. In 8/36 subjects (22.2%), we decided to scale up the therapy within the next 12 months from transition (Table 2).

**Table 2:** Transition-related outcomes

Total no. of patients	36 (100%)
JADAS-27 score before transition, median (*Q*1–*Q*3)	2 (0–5.15)
JADAS-27 score after transition, median (*Q*1-*Q*3)	0 (0–0)
Reduction in JADAS-27 after transition, *n* (%)	8/36 (22.2%)
Change in therapy after transition, *n* (%)	22/36 (61.1%)
Need of step-up therapy, *n* (%)	8/36 (22.2%)
Lost to follow-up	0/36 (0)

### Comparison between patients with and without need for step-up therapy

In the eight patients who needed to scale up the therapy within the next 12 months from transition, no correlations were found with the JIA subtype, age at onset, type of involved joints, and all the other variables mentioned above. We found a major prevalence of tenosynovitis in such patients, although no statistical significance was reached (Table 3).

**Table 3:** Comparison between patients with the need for scale-up therapy and those with controlled diseases after transition

	Scale-up therapy	Controlled disease	***p***-value
No. of patients (%)	8/36 (22.2%)	28/36 (77.8%)	
Female sex, *n* (%)	5/8 (62.5%)	21/28 (75.0%)	0.66
Family history of autoimmunity, *n* (%)	2/8 (25.0%)	4/28 (14.3%)	0.60
JIA categories^1^, *n* (%)			
Polyarticular	2/8 (25.0%)	7/28 (25.0%)	
Oligoarticular	4/8 (50.0%)	9/28 (32.1%)	
Psoriatic arthritis	1/8 (12.5%)	7/28 (25.0%)	0.83
ERA	1/8 (12.5%)	2/28 (7.1%)	
Systemic	0/8 (0.0%)	3/28 (10.7%)	
Polyarticular and oligo-extended	5/8 (62.5%)	11/28 (39.3%)	0.42
Other than oligo-extended, *n* (%)	3/8 (37.5%)	17/28 (60.7%)	
Oligoarticular *n* (%)	1/8 (12.5%)	5/28 (17.9%)	1.00
Other than oligoarticular	7/8 (87.5%)	23/28 (82.1%)	
Cervical spine involvement, *n* (%)	2/8 (25%)	4/28 (14.3%)	0.60
Enthesitis, n (%)	1/8 (12.5%)	5/28 (17.9%)	1.00
Tenosynovitis, *n* (%)	3/8 (37.5%)	3/28 (10.7%)	0.11
Uveitis, *n* (%)	2/8 (25%)	3/28 (10.7%)	0.31
ANA positivity, *n* (%)	5/8 (62.5%)	9/28 (32.1%)	0.22

^1^JIA categories according to 2001 ILAR classification criteria

### Likert scale, results of open questions

When directly asked, through the online survey, 83.3% (15/18) of patients declared they were satisfied or very satisfied with the transition process, while 16.7% (3/18) declared they were not (response rate 50%). The most frequent reason for satisfaction was “the presence of the referral pediatrician in the adult hospital setting during the first transition visits,” which was perceived as a safe element of continuity. Also “the central geographical position of the adult referral center” was appreciated. On the other hand, “addressing several physicians other than rheumatologist for different health-related problems” was the main complaint of young adults with JIA, who preferred to report all their health-related problems to a medical team in a unique setting.

## Discussion

The transition model discussed in this study resulted in good transition outcome measures, as shown by the fact that all patients adhered to follow-up visits, by the significant reduction of JADAS-27 score after completing the transition, and by the high level of satisfaction reflected by the answers of the survey. The number of patients maintaining regular appointments with adult rheumatology is higher in our study (100%) than what is reported in literature (75%), and the same applies to the response rate to the survey (50% in our study) which is about 34% in literature for patients who were asked to complete a mail questionnaire [9, 20, 21]. Also, although the period of follow-up after the transition was limited, we have longer follow-up data for a minority of patients which confirms the results on absent drop-outs. Furthermore, we performed the first transition visit in the adulthood health care setting 114 days after from the last pediatric visit (SD 132) which is soon if compared with literature (221 days) [22].

In our study, 22.2% of patients needed a scale-up therapy, which indirectly means that they had an active disease during the transition phase. This percentage is lower than what was reported in the study by Foster et al., where 39% of adult patients with JIA (median age 30), who did not undergo a specific transition program, had an active joint disease (according to the physical global assessment scale of disease activity, PGA). In the same study, patients also showed low scores in health-related quality of life measures, explored through the Short Form (SF)−36 questionnaire when compared with controls, and this was irrespective of the degree of functional disability [23]. Such findings support the idea of Schmidt et al. according to which a structured adult health care transition intervention (i.e., a structured transition program including the steps of planning, transfer, and integration) regardless of what is associated with better outcome measure at any level (quality of life, adherence to care, disease-activity measures, satisfaction rate, etc.) [24].

However, our results also differ from those shown by Scagnellato et al., who examined risk factors for relapse in a cohort of patients with JIA after transition to adult care [25], showing a 46% relapse rate in a cohort of 50 patients undergoing transition between 16 and 18 years old. Their program included discussion with families about transitioning upon turning 18 years old, a preliminary meeting between pediatric and adult rheumatologists (caring for the patient at least for 3 years), a joint visit with the patient and family, along the presence of a dedicated nurse. This suggests that the type of transition program can lead to very different results, with other factors affecting the outcome measures. Also, in our cohort, there was no correlation between JIA features and the other variables examined among the eight patients who needed a scale-up therapy after transition. Our patients had respectively polyarticular (2), oligo-extended (3), psoriatic (1), and enthesitis-related arthritis (1), with only one presenting oligoarticular JIA. Such results are different from what was reported by Scagnellato et al., who found a higher frequency of monoarthritis among relapsers instead. Although not statistically significant, we observed a trend correlation between major tenosynovitis and the need for scale-up therapy, suggesting that the presence of tenosynovitis in pediatric age could be an early marker of disease relapse after transition. In this regard an important diagnostic/prognostic role could be played by musculoskeletal ultrasonography [26], which is able to early detect tendon inflammation, thus allowing pediatric patients to be recognized as at-risk individuals with poorer transition outcomes. Indeed, the prognostic role of tenosynovitis on relapse risk after transition needs further investigation.

Furthermore, reasons for changing therapies varied from using treatments approved for adults but not for pediatric patients to adjusting therapies, to provide better disease control according to a different adult-type approach. Only one patient of the entire cohort had polyarticular JIA with positive rheumatoid factor (RF) and anti-citrullinated protein antibodies (ACPA). The patient was then re-classified as being affected by rheumatoid arthritis and showed good disease control by therapy.

Further studies are needed to explore which factors are more associated with poor transition outcomes in adult age and if such factors should be attributed more to the biological mechanisms of the disease or to psychosocial vulnerabilities; the family, in this regard, has a very strong impact on the success of the process. Disease relapse is considered a measure of poor transition outcome, along with the need for enhancing the ongoing therapy for poorly controlled disease. However, the fact that the majority of our patients showed good disease control and that nobody was lost during the follow-up is an indication of good adherence to therapy leading to good transition outcomes.

This study shows the transition experience of a cohort of patients with JIA in the Friuli Venezia Giulia region of Italy. Our Institute is a tertiary referral pediatric university teaching hospital, serving an area of 250,000 people with 35% patients coming from outside the Trieste and Udine provinces. In such a context, the more central geographic position of Udine, where the adult rheumatology referral center is located, could have contributed to a successful transition process [6], as confirmed by the answers of the patients. The multidisciplinary approach of adulthood medicine, which consists of addressing multiple specialists, each one taking care of a single aspect, is the strongest negative reason for dissatisfaction reported by patients and their families. There is no question that, for proper care of both pediatric and adult patients, different professionals are needed to meet different medical requests. However, the more integrative pediatric approach, with the pediatrician acting as a case manager, facing different medical problems of the patient, and sharing opinions with other specialists, is not applicable to adulthood health care. Since adult rheumatologists cannot afford all the medical issues raised with age, the only way to reduce this gap is by enhancing communication between adulthood specialists and reducing bureaucracy.

This study builds on a transitional model based on a few core elements, chosen among the ones reported in literature [6, 7, 15] for their feasibility and consistent recurrence throughout all the transitional models proposed. They can be summarized as follows: “focusfirstonadolescent mental health,” “early discussoftransition with patient and families,” “involve the patient in decision making over time,” and “provideshared clinical appointmentswithboth pediatric and adult physicians.” We think that the main point of strength of our approach is based on a patient-centered model of care which focuses on adolescence first. Reinforcing self-awareness, encouraging positive coping strategies, ensuring a functional peer group, and minimizing catastrophism have been recognized as crucial elements to improve adolescents’ health-related quality of life [2]. About 10–15% of teenagers reported that chronic conditions impact their lives not only because of the disease activity itself, but also because their clinical condition generates anxiety, depression, disease cognition, and a sense of loneliness [2, 27]. In our experience, what has emerged from the early discussions with the adolescents during their transition process is that they want to be acknowledged and addressed. They want to express and overcome their fears and are interested in learning more about their disease and its consequences. Considering the developmental trajectories of adolescence and the social and economic burden of bad related outcomes, facing transition should be a primary point on the health system agenda and a crucial field where to invest for the future of young people with chronic disease [28].

Limitations of this study include the small sample size, since the low number of patients limits the interpretation of results. In addition, none of the questionnaires available in the literature for assessing patients’ readiness to transition was used [29–31]. Nevertheless, we think the continuous involvement of the patients during the visits according to the way out transition policy is organized, could overcome this limitation. Then, a semi-structured self-made survey composed of a 5-point Likert scale [19] and two open questions was used in this study. Although not validated in the literature, this approach was made to minimize the time needed to fulfil the survey and to obtain a higher response rate. Actually, about 50% of the patients did answer the questions, which is more than what was reported in the literature for similar experiences [9, 20].

## Conclusions

In conclusion, this monocentric study shows good results in terms of transition-related outcomes, as all patients adhered to follow-up visits, and the majority showed a reduction of JADAS-27 and a high level of satisfaction score after completing the transition process. We think that a model of care which focuses first on adolescence, with its neurophysiological specificity as a crucial developmental and motivational age, makes the transition process a natural consequence of a physiological change. This way of continuously addressing patients will also warrant them a better memory of the disease, as well as those who will never be transitioned to adult age because of remission occurring within their pediatric age. Future research is needed to confirm such an assumption and a direct comparison of this transition model with other transition models is warranted.

## Supplementary information

Below is the link to the electronic supplementary material.

ESM 1 (DOCX 14.5 KB)

## Data Availability

The data that support the findings of this study are available from the corresponding author, S.P., upon reasonable request.
