# Supplementary material for: Transition-related outcomes among a cohort of patients with juvenile idiopathic arthritis
Source: Clin Rheumatol. 2025 Jan 16;44(3):1377–84. doi: 10.1007/s10067-025-07317-y (PMC11865105; doi:10.1007/s10067-025-07317-y)
Supplement: Supplementary file 1 — (DOCX 14.5 KB) [file 10067_2025_7317_MOESM1_ESM.docx]

**Supplementary file 1.**

**Survey on patients’ satisfaction about transition process**

1. **How much do you feel satisfied about your transition process from pediatric to adult care?**

❏ Very satisfied

❏ Satisfied

❏ Neutral

❏ Unsatisfied

❏ Very unsatisfied

1. **Which are the aspect you are more satisfied with? (Open question)**
2. **Which are the aspect you are not satisfied with? (Open question)**
